# A case of Cushing Syndrome due to accidental intake of dexamethasone: a call for enforcement of regulatory laws

**DOI:** 10.4314/gmj.v55i2.9

**Published:** 2021-06

**Authors:** Kofi T Asamoah, Josephine Akpalu, Yacoba Atiase, Ernest Yorke

**Affiliations:** 1 Department of Medicine & Therapeutics, Korle-Bu Teaching Hospital, Accra, Ghana; 2 Department of Medicine & Therapeutics, University of Ghana Medical School, Accra, Ghana

**Keywords:** Traditional medicine, Cushing syndrome, corticosteroids, misuse of orthodox medication, drug regulation enforcement

## Abstract

Cushing syndrome could be a complication of long-term steroid use, resulting in a wide range of clinical presentations. Whereas some patients take medically prescribed doses of conventional medicines, including steroids for various ailments, others inadvertently consume unspecified doses by adulterating traditional medicines with these orthodox medications to increase their therapeutic appeal. Furthermore, some individuals with access to conventional medications may sell them in unlabelled packages as traditional medicines (TMs) to unsuspecting customers. This may lead to undesirable side effects and safety concerns. The case report highlights the problem of poorly regulated access to medications and makes suggestions to protect patrons' health of traditional medicines.

**Funding:**

None declared

## Introduction

The use of traditional medicines (TMs) is widely considered safe and efficacious by citizens of developing countries^1^-^4^ largely due to the belief that they are produced entirely from natural sources.^4^ Though some studies find that patronage is more among rural folk with poor access to modern healthcare services^4^, other authors found that highly educated people with good incomes also patronize TMs.^1^,^5^ Some TMs are, however, adulterated with conventional medications to widen their therapeutic coverage and appeal.^3^ This may alter their safety profiles, which are already poorly understood^1^, and increases the risk of experiencing side effects.^3^,^5^ Unsuspecting patrons, due to their trust in TMs, may purchase conventional drugs being marketed as TMs by individuals with access to them. These may be taken at medically harmful doses, putting the patron at risk.

We present a report on a patient who developed Cushing syndrome, a well-known complication of chronic steroid use following his patronage of a drug he thought was a TM. This report highlights the need for stricter regulations governing conventional drugs and those purported to be TMs to protect the public from potential profound negative health consequences.

It is also expected that the report will engender public awareness and discussions on the subject, which will eventually lead to policy reforms and enforcement to combat the menace.

## Case Report

The patient gave his consent for the use of his information and pictures for the publication,.

A 42-year-old Ghanaian male trader presented with increasing fatigue, increased appetite, increased body weight, polyuria, nocturia, repeated skin infections and muscle weakness, especially climbing stairs and washing his hair. These symptoms had lasted for over a year after taking purported TMs purchased from a bus terminal. He had also been diagnosed with hypertension a year earlier and was prescribed amlodipine but stopped taking it because his condition did not improve. He preferred taking TMs because it made him feel better. He also felt worse whenever he attempted stopping and consequently needed help with normal daily living activities.

He was asked to do an abdominal computed tomography (CT) scan following an unspecified complaint at a visit to his primary healthcare facility.

The scan showed hepatomegaly, increased peritoneal fat and a diaphragmatic hernia containing fat. Following this, he was referred to the Korle Bu Teaching Hospital for further management.

**Figure 1:**
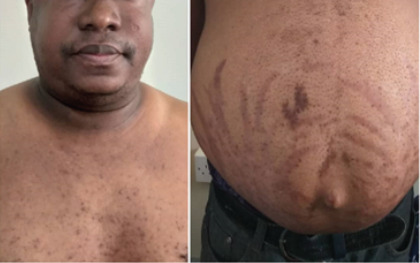
Pictures showing moon face, acneiform eruptions on the chest and striae on the abdomen

**Figure 2:**
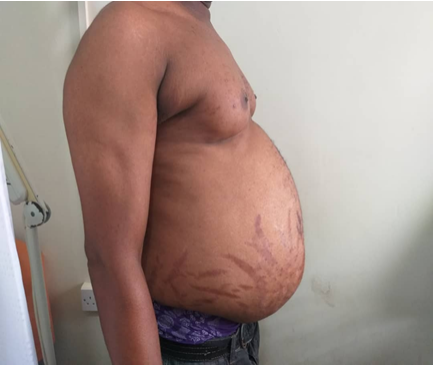
Lateral view of the patient's chest and abdomen showing pseudo-gynaecomastia and striae on the abdomen

Physical examination showed he was obese (BMI of 32kg/m^2^) with a moon face, a buffalo hump, acneiform eruptions on his face and chest. He also had pseudo-gynaecomastia, thin arms compared to his trunk, and florid wide, purple striae on his abdomen (Figures 1 and 2). His blood pressure was 143/93mmHg, with a regular pulse of 120 beats per minute. There was evidence of mild proximal muscle weakness.

Laboratory investigations requested showed a fasting blood sugar of 5.6mmol/L (normal), glycated haemoglobin of 6.7% (elevated), and 08:00 hours serum cortisol was 8umol/L (low). Blood urea, electrolytes and creatinine were all within normal ranges. An electrocardiogram showed left ventricular hypertrophy, while an echocardiogram revealed hypertensive heart disease with a reduced ejection fraction of 26%.

**Figure 3:**
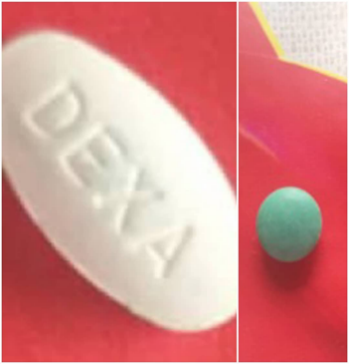
Pictures of traditional medications purchased by the patient from a traditional healer

On request, he presented with his TM. These were two pills: a green one of unknown composition and a white one with a “DEXA” inscription (Figure 3). The strength was, however, unknown.

Given his symptoms, signs, low 8 AM serum cortisol level and intake of the DEXA tablet, the following diagnoses were made: Exogenous Cushing syndrome and Hypertensive heart disease with a reduced ejection fraction

He was commenced on carvedilol 12.5mg daily, furosemide 40mg bd, lisinopril 10mg daily and tapering doses of prednisolone and counselled about the content of his TMs and its possible relationship with his current presentation. A dietician visit was arranged because of his hypertensive heart disease, and an oral glucose tolerance test (OGTT) was requested due to the discordant FBS and HBA1c results. The OGTT was, however, not done. He reported improved exercise tolerance and an improved sense of general well-being after his third follow up visit, but unfortunately, the patient was lost to follow up despite efforts to get him to return for a review.

## Discussion

Cushing syndrome is a rare condition caused by hypercortisolism characterized by phenotypic changes in fat distribution, myopathy, skin and metabolic changes.^6^ In subclinical hypercortisolism, clinical features are absent or very mild, with biochemical evidence of elevated cortisol.^7^,^10^,^11^

The commonest cause is iatrogenic (exogenous), due to the use of steroids in managing chronic inflammatory, neoplastic and autoimmune conditions.^7^,^9^

Though oral steroids are the most frequent culprits, all other routes, including inhaled, topical, intramuscular, are implicated in the development of Cushing syndrome.^7^,^12^ Diagnosis of exogenous Cushing syndrome is dependent on a good clinical history to identify potential exogenous sources of glucocorticoids.^13^ Endogenous Cushing syndrome, mainly due to tumours of the pituitary and adrenal glands, is treated surgically.

The use of TMs is rising exponentially worldwide.^2^,^14^ A study in Malaysia showed that up to 50% of citizens with chronic illnesses patronized TMs^5^, while Ugandan studies report a prevalence of about 60%.^1^,^2^ TMs are patronized for various reasons; cultural beliefs of safety and efficacy despite a dearth of scientifically proven information, a recommendation from friends and family, inaccessibility to or frustration with conventional medical services, lower cost than orthodox medicine, among others.^1^,^2^,^4^,^5^ Only a few TMs go through rigorous testing for safety, posing a potential risk to patrons.^14^ Some of these medicines are sold in pouches, with no information on dosing, composition or expected side effects.^15^ The patient presented in this paper intended to purchase TMs but unwittingly ended up with oral dexamethasone. His supplier was in direct contravention of the Public Health Act of Ghana (Act 851, 2012), which states specifically that it is an offence to adulterate drugs, incorrectly label and misinform about therapeutic value.^16^ This is especially a problem because our patient may just have been one of many others who have patronized those services.

Though unlawful to advertise a product as a cure for chronic ailments and subfertility as stated in the 5th Schedule of the Public Health Act 851,^16^ TMs are often marketed as a panacea for these, including hypertension.^1^,^5^ TMs are widely available in Ghana, with some being sold on public transportation, by the roadside, in pharmacies or even prepared at home. TMs and some orthodox medications are purchased without prescriptions. This gives many people unrestricted access to them, another regulatory lapse that must be addressed, as these orthodox medications can be repurposed in any way deemed fit by the buyer. Ching et al. found that adulteration of TMs with other chemicals was quite common, with steroids as the third commonest contaminant found in proprietary Chinese medicines in a study in Hong Kong.^3^ A similar observation was quoted by Sazlina et al. in a Malaysian report, with other common contaminants being weight-loss drugs, oral hypoglycaemic agents and non-steroidal anti-inflammatory drugs.^5^ Tong and Rajoo reported on a patient who also developed Cushing syndrome after taking proprietary Chinese medicine known to contain glucocorticoids.^13^ These all highlight the misuse of orthodox medications by individuals trusted to supply TMs to unsuspecting clients.

The perception that medical practitioners disapprove of the use of TMs 2,5,14 affects patients' disclosure of their use of TMs. James et al. found that there was a non-disclosure rate of up to 83%.^4^ This is somewhat corroborated by our patient's failure to disclose and present his drugs until the attending physician expressly asked him. It is therefore important for doctors to accept that TMs are rife^12^ and specifically ask about their usage.^5^,^13^

### Policy reforms and enforcement

The integration of traditional and complementary medicines into the medical school curriculum to educate doctors more of TMs and their safe use, backed by scientific research, is highly recommended.^2^, ^4^ This is particularly important because identifying the TM and its constitution may be all that is required to make a diagnosis, as in this case. Furthermore, concomitant use of TMs and orthodox medicines could increase the frequency of adverse reactions due to potential drug interactions.^1^

TMs are less likely to be subjected to quality control measures including post-marketing surveillance, according to James and Ekor.^4^,^14^ Adequate surveillance will assist in detecting wrongful sale of medications under the guise of others, which could have curtailed this patient's exposure. Local law enforcement agencies and task forces set up by regulatory authorities therefore need to hold TM producers and merchants to the same standards as their counterparts who deal with orthodox medicines, as stated in the national laws. Ghana's Public Health Act (Act 851, 2012) spells out clear standards to be followed^16^ and contraventions must be punished appropriately. Medical practitioners also need to play an active role in monitoring and reporting any related unlawful activity to the regulatory authorities.

Allocation of resources for research into the appropriate formulation and dosage of TMs will help ensure that the best is being offered to the public and give them confidence that these medications, to which they have cultural ties, are being acknowledged. This may impact positively on adherence.

Efforts on public education also need to be improved, targeting both the producer and consumer. Producers and merchants need to be aware of the standards to which they are being held and ways to improve their output based on modern Science and Technology, as well as the consequences of flouting the laid-down rules of practice. Consumers must be empowered on these medications, particularly their spectrum of activity and considerations before purchase, to safeguard their own health.

## Conclusion

Individuals with access to medications may repurpose them any way they desire, potentially putting health consumers at risk as seen in this case. This calls for vigilance by all stakeholders to safeguard the health of the public.
